# Treating Lower Phantom Limb Pain in the Postoperative Acute Care Setting Through a Virtual Reality-Based Graded Motor Imagery Program: A Case Series

**DOI:** 10.1177/29941520251410666

**Published:** 2025-12-29

**Authors:** Marinya Roznik, Megan Crooks, Michael Smith, Elizabeth Hammond, Patrick Gross, Thomas Mutter, Renée El-Gabalawy

**Affiliations:** 1 Department of Anesthesiology, Perioperative and Pain Medicine, University of Manitoba, Winnipeg, Canada.; 2 Department of Psychology, University of Manitoba, Winnipeg, Canada.; 3 National Research Council of Canada, Winnipeg, Canada.; 4 Department of Physical Therapy, University of Manitoba, Winnipeg, Canada.; 5 Shared Health Manitoba, Manitoba, Canada.; 6 Department of Clinical Health Psychology, University of Manitoba, Winnipeg, Canada.; 7 Department of Psychiatry, University of Manitoba, Winnipeg, Canada.; 8 CancerCare Manitoba, Winnipeg, Canada.

**Keywords:** phantom limb pain, lower limb amputation, virtual reality, graded motor imagery, postoperative, case series

## Abstract

**Background:**

Phantom limb pain (PLP) affects over 70% of people with lower limb amputations (LLAs). Graded motor imagery (GMI) is an established nonpharmacological treatment that targets the cortical mechanisms associated with PLP onset and may be the most effective if it is administered shortly following amputation. However, application of GMI in practice is hindered by the need for trained health care professionals to assist with longitudinal administration and by low patient buy-in (i.e., one’s understanding of, and motivation to engage in, a treatment modality). To address these barriers, our research team developed a virtual reality (VR) program to facilitate self-administration of GMI.

**Objectives:**

The primary objective of the current case series was to assess initial feasibility of using the VR GMI program in the acute postoperative setting by evaluating recruitment trends. The secondary objective was to describe the satisfaction, usability, and tolerability of the VR GMI program after short-term administration.

**Methods:**

Patients undergoing LLAs at a single academic tertiary hospital were screened for eligibility and interest, and consenting individuals underwent daily sessions in the VR GMI program. Adverse events (i.e., any instance in which pain or nausea interrupted VR engagement, as indicated by the Numerical Rating Scale) were monitored to evaluate tolerability. A self-report questionnaire assessed satisfaction and usability.

**Results:**

Of 52 individuals screened, 41 were not eligible to participate (79%). Five eligible individuals were not interested in participating due to feeling overwhelmed (10%). Six participants were recruited (12%), and four were able to complete the entire intervention before their hospital discharge (8%). Most participants rated the program as easy to understand, enjoyable, and helpful. However, participant scores also indicated the need to improve usability. Only one participant reported an adverse event (i.e., fatigue in phantom limb).

**Conclusions:**

These results will directly inform refinement of the VR GMI prototype and related VR research implemented in the postoperative setting.

## Introduction

Over 7000 Canadians undergo lower limb amputations (LLAs) each year, of which approximately 70% will experience phantom limb pain (PLP)—a form of neuropathic pain perceived in a limb despite its physical absence following amputation.^1,2^ PLP onset can occur within the first few days to 1 month following amputation, with most people with LLAs experiencing it within 1 year.^3^ Recent studies also suggest PLP may be the most amenable to intervention within the first 6 months following amputation, theoretically by preventing the maladaptive reorganization of the sensorimotor cortex implicated in PLP.^4–6^ One such intervention, graded motor imagery (GMI), was originally developed by Moseley and colleagues as a structured, sequential protocol designed to progressively restore sensorimotor congruence and reduce pain in conditions such as PLP and complex regional pain syndrome.^7^ GMI comprises three distinct stages: (1) implicit motor imagery, achieved through a left/right limb discrimination task; (2) explicit motor imagery, involving the mental simulation of limb movements without overt execution; and (3) mirror therapy, which stimulates phantom motor imagery through visual illusion created by positioning the intact limb in front of a mirror. Meta-analyses indicate that GMI is an efficacious intervention compared with placebo or its individual components, such as mirror therapy or explicit motor imagery alone, and findings from an individual randomized controlled trial further suggest that adherence to the protocolized sequence of stages is essential for reducing PLP intensity according to validated self-report measures.^8–10^ Unfortunately, effective intervention is often delayed due to long outpatient wait times (>6 months in Canada^11^), the intensive involvement of health care personnel required for longitudinal administration,^12^ limited patient motivation for or belief in the efficacy of GMI (also known as treatment buy-in),^13^ and an overreliance on pharmacological approaches that have demonstrated limited effectiveness for PLP.^8,14^ Given PLP has been independently associated with lower quality of life, as well as increased rates of depression, anxiety, and suicidality, improving the timeliness and accessibility of effective interventions is warranted.^14^

The feasibility of GMI may be improved through virtual reality (VR)—a three-dimensional computer-generated simulation in which a user is immersed in a virtual world through a headset.^15^ Recent systematic reviews suggest that virtual visual feedback of the phantom limb, mimicking mirror therapy, is an efficacious PLP treatment in community settings.^15,16^ Phantom motor execution—characterized by the control of a virtual phantom limb through electromyographic signals detected at the residual limb—has likewise demonstrated efficacy for PLP treatment and comparable outcomes to explicit motor imagery.^17^ Case series also indicate that VR-based treatments, incorporating both virtual visual feedback and phantom motor execution, can be successfully self-administered at home and show preliminary effectiveness.^18–20^ Studies further suggest that the immersion and gamification offered by VR make treatment more engaging and may enhance compliance, even in hospital settings.^21,22^ However, nearly all existing studies have been conducted months or years after amputation in chronic pain populations, leaving a critical gap in understanding how VR-based interventions could be implemented during the immediate postoperative recovery period—when cortical reorganization is most dynamic and potentially modifiable.^3–6,15^ While mirror therapy, explicit motor imagery, and phantom motor execution have each been tested through VR, the sequenced GMI protocol has never been implemented, despite its potential to improve accessibility through self-administration and gamified delivery. VR has shown feasibility in nonsurgical hospital settings, typically over one to five sessions for distractive pain relief, suggesting that inpatient delivery is both possible and acceptable.^22^ However, the feasibility of VR-based GMI as an early PLP intervention remains unknown.

The present proof-of-concept study aims to evaluate the feasibility of administering a VR GMI program during the immediate postoperative period following LLA. It represents the next phase in a staged program of research guided by the Virtual Reality Clinical Outcomes Research Experts (VR-CORE) model and follows an earlier developmental phase in which the VR GMI program was refined through input from people with lived experience of LLAs who trialed the prototype and provided feedback to enhance its usability and interactivity.^23–25^ In accordance with the VR-CORE model, this phase represents an early feasibility evaluation intended to refine methodological procedures and inform the design of a subsequent, larger-scale investigation. The primary objective was to assess the feasibility of the VR GMI program in the acute postoperative setting via recruitment, eligibility, and consent rates; the secondary objective was to explore patient satisfaction, usability, and tolerability with the VR GMI program following short-term use during postoperative recovery.

## Methods

The current single-center prospective case series was approved by the Bannatyne Research Ethics Board at the University of Manitoba (B2022107). It was conducted between January 2024 and July 2024 at the Health Sciences Center (HSC) in Winnipeg, Canada. The HSC is an academic tertiary hospital and level I trauma center where LLAs are performed by orthopedic surgeons, vascular surgeons, and orthopedic oncologists. Methods and materials pertaining to the development of the VR GMI program are outlined in detail in a previous protocol and are summarized in this section.^23^

### Recruitment

Potential participants were recruited in both the preoperative and immediate postoperative periods. They were identified by daily screening of preanesthetic clinic referrals and elective and emergency operating room slates and by reviewing eligible admissions to surgical wards through weekly phone calls with nursing staff. Potential participants were required to fulfill the following inclusion criteria: (1) be scheduled for primary or revision unilateral LLA or have undergone a primary or revision unilateral LLA (above knee, below knee, or midfoot amputation) within the last 5 days, (2) be at least 18 years old, and (3) be able to speak and read English fluently. Participants were excluded if major bilateral amputations (i.e., proximal to the midfoot) were planned, a prior contralateral amputation proximal to the midfoot existed, or if visual, hearing, cognitive, or motor impairment would affect engagement with the VR headset. After identifying a potential participant, a member of the patients’ health care team would approach the individual to gauge interest in speaking with the research team about the VR study. If interested, a member of the research team would then confirm eligibility and obtain informed consent.

Two amendments were made to improve study procedures following the start of recruitment in response to insufficient enrollment rates. First, within 1 month of study initiation, the eligibility criteria were expanded to include individuals who had undergone an LLA within the past 2 months (rather than the past 5 days) in line with emergent literature.^4^ This broader window allowed the research team to capture participants who remained hospitalized long enough to be approached and complete the intervention, rather than being discharged before contact could occur. Individuals were also excluded if they were discharged or transferred from HSC within 3 days of their amputation, as they could not realistically complete the full VR GMI program within that timeframe, or if nursing staff recommended exclusion due to clinical necessity. Second, during the final 2 months of recruitment, study personnel began consulting weekly with health care team members on the surgical units (e.g., charge nurses) to support recruitment. These procedural adjustments were critical to overcoming early recruitment barriers and reflect the iterative nature of novel feasibility research.

### Intervention

The VR GMI program consisted of three sequential stages—left/right discrimination, explicit motor imagery, and limb simulation—aligned with the traditional GMI protocol.^7^ In the left/right discrimination stage, participants completed a series of laterality judgments across progressively challenging levels, identifying whether displayed limb images were left or right until all levels were completed, typically lasting about 10–15 min.

In the explicit motor imagery stage, participants completed two guided audio recordings: a body-scan promoting awareness of the body and a meditative imagery exercise directing attention from the intact to the phantom limb. This stage typically lasted 20–30 min.

The final limb simulation stage provided visual–motor feedback through a life-size virtual self-avatar embodied from a seated position within a simulated hospital room environment, mirroring the inpatient context. Participants observed their avatar performing slow, symmetrical limb movements while imagining and executing the same actions with their phantom limb. Sessions in this stage generally lasted 15–20 min but were adapted based on fatigue and comfort.

Tasks were lightly gamified to sustain engagement, and participants typically advanced one stage per day after meeting minimum task criteria without adverse events (*defined in the Procedures section*). The program was delivered bedside using an Oculus Meta Quest 2 headset, with progress mirrored on a laptop using an application called SideQuest to allow the research assistant (RA) to observe in-headset activity, troubleshoot technical issues, and provide real-time verbal scaffolding. More details on the VR GMI program, as well as visual illustrations, can be reviewed in a previous publication.^23^

### Procedure

Following informed consent, sociodemographic information was collected via interview. Prior to each VR GMI session, participants were given the option to refuse the daily session; reasons for refusal were recorded. The RA provided detailed verbal instructions on how to use the VR program. Once the participant expressed assent to proceed, the RA assessed preintervention nausea, PLP, and residual limb pain (RLP; i.e., physical pain at the amputation site) through verbal administration of the Numerical Rating Scale (NRS; 11-point Likert scale validated for pain and nausea^26,27^). Nausea was measured because it is a common symptom among hospitalized patients and a known side effect of VR use.^28^ RLP was also assessed because it is highly correlated with PLP and often conflated with it in measurement, enabling the research team to distinguish whether participants’ reported discomfort stemmed from neuropathic (phantom) or somatic (residual limb) sources.^2^

The participant could enter one VR GMI stage per day and was only permitted to progress to the next stage in the following session if they did not experience any adverse events (their NRS scores for PLP, RLP, or nausea did not increase by more than two points) during the previous session. The rationale for this choice is that GMI treatment can worsen PLP if progression is too rapid.^7,29^ Participants could engage with the VR GMI for as long as they wished in a single session. After each session, the NRS for PLP, RLP, and nausea was readministered to capture pre- to post-VR changes and determine whether any discomfort or nausea emerged specifically during VR exposure, rather than reflecting the natural course of postoperative recovery. The RA documented any impromptu feedback as observational field notes. Intervention sessions were concluded if any of the following criteria were met: the participant was discharged or transferred from the HSC; the participant chose to withdraw from the study; and the participant progressed through all three of the VR GMI stages.

After the final VR GMI session, the VR Impressions Scale was administered, a quantitative and qualitative measure developed and verbally administered by the research team to evaluate satisfaction with and usability of a VR program.^23,30^ It primarily consists of seven statements that participants indicate their agreement with on a scale of 0 (*strongly disagree*) to 100% (*strongly agree*). For example, participants respond to the statement, “I enjoyed my session with the VR system.” Participants also had the opportunity to respond to open-ended questions regarding what they liked and disliked about the program, as well as whether they found the VR GMI program to be worthwhile. Although the VR Impressions Scale has been employed in prior feasibility studies, it has not yet been psychometrically validated; therefore, results derived from this measure should be considered exploratory and interpreted with caution.^30,31^

### Analytic strategy

Descriptive statistics were used to analyze recruitment trends. Raw quantitative data collected from the VR Impressions Scale were presented as percent endorsement (0–100%) of each questionnaire statement and reported for each individual participant. An endorsement of ≥50% was considered to indicate agreement, in line with qualitative anchors. NRS scores were flagged to reflect a clinically significant increase in PLP, RLP, or nausea in any given VR GMI session if the pre–post change in the raw scores were ≥3.

## Results

### Objective 1: Recruitment trends

**FIG. 1.**
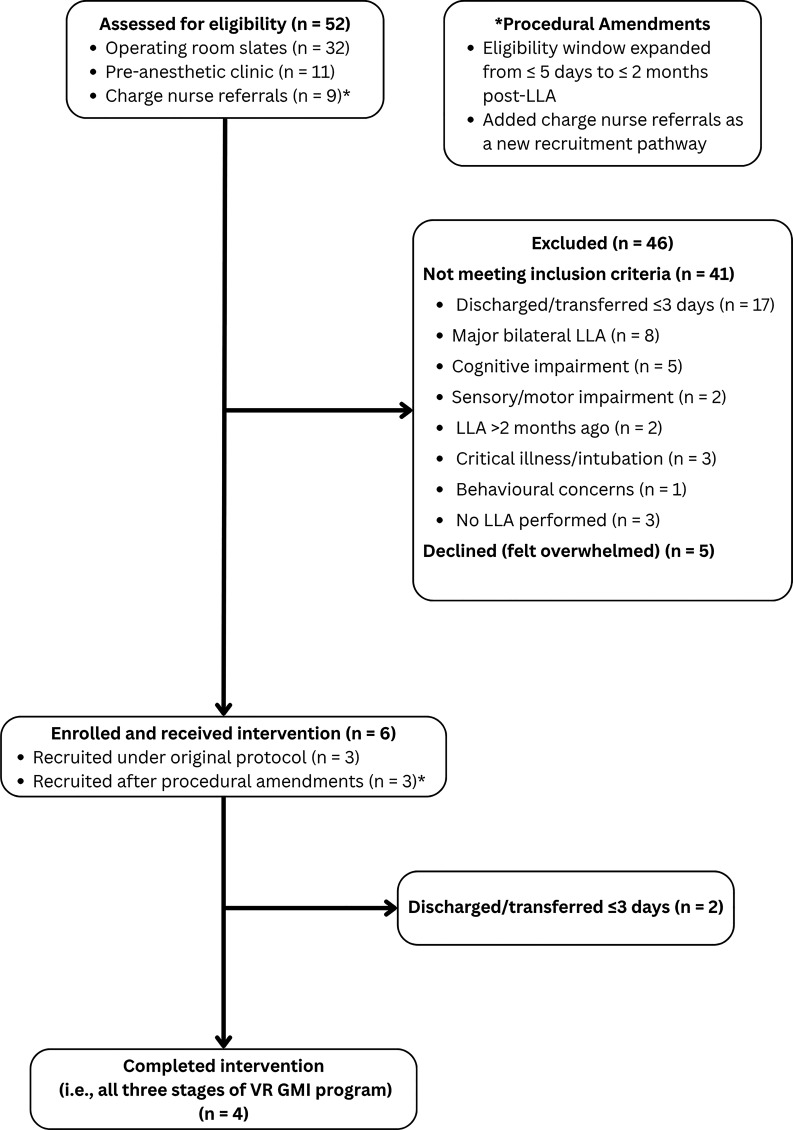
CONSORT flow diagram illustrating participant screening, enrollment, and completion of the VR GMI program over a 7-month recruitment period (January–July 2024). Of 52 individuals assessed for eligibility, 6 were enrolled and received the intervention, and 4 completed all three stages of the VR GMI program. Asterisk (*) indicates procedural amendments made mid-study that expanded eligibility criteria and added charge-nurse referrals as a new recruitment pathway. CONSORT, Consolidated Standards of Reporting Trials; LLA, lower limb amputations; VR GMI, virtual reality graded motor imagery.

Over the 7-month recruitment period (January 2024–July 2024), 52 LLA recipients were screened by the research team. Thirty-two were identified through the operating room slates (62%), 11 were identified through the preanesthetic clinic (21%), and 9 were identified through weekly correspondence with charge nurses in the last 2 months of recruitment (17%). Of these, six consented to participate (12%), and four completed all three stages of the VR GMI program. Two of the six consented participants (33%) were discharged before completing the program. Three participants were recruited through health care team members on the surgical units within the last 2 months of recruitment, whereas the other three were recruited through the operating room slates or preanesthetic clinic within the first 5 months. Three participants began the VR program more than 5 days after their amputation following the expanded eligibility window, whereas the other three were recruited within the original 5-day timeframe. Participant flow through screening, eligibility, and intervention completion is presented in Figure 1, following the structure of the Consolidated Standards of Reporting Trials 2010 Statement adapted for a case series design.^32^

**FIG. 2.**
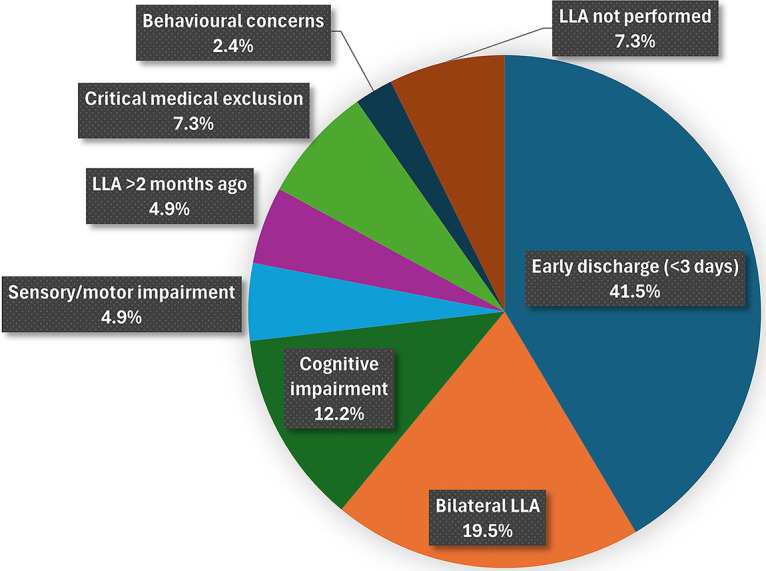
Pie chart illustrating various reasons for exclusion from trialling the VR GMI program (*n = 52*). LLA, lower limb amputation; VR GMI, virtual reality graded motor imagery.

Of the 52 screened individuals, 41 were deemed ineligible to participate (79%), and 5 declined participation despite meeting eligibility criteria due to feeling overwhelmed by receiving a LLA (10%). The remaining six (12%) consented and began the intervention. Among the 41 ineligible individuals, the most prevalent reasons for exclusion included discharge or transfer from the hospital within 3 days following their LLA (*n =* 17, 33%), major bilateral LLA (*n* = 8, 15%), cognitive impairment (such as delirium or impaired level of consciousness; *n* = 5, 10%), sensory/motor impairment (including severe diabetic retinopathy/neuropathy and hearing difficulties; *n* = 2, 4%), and receiving an LLA or revision over 2 months ago (*n =* 2, 4%). Other reasons for exclusion not included in the initial eligibility criteria were medical conditions that prevented VR engagement, such as critical illness or intubation (*n* = 3, 6%), and behavioral concerns expressed by the health care team (*n* = 1, 2%). Three individuals were screened but ultimately did not receive an LLA (*n* = 3, 6%). See Figure 2 for a pie chart summarizing the various reasons for exclusion.

### Objective 2: VR GMI program impressions

**FIG. 3.**
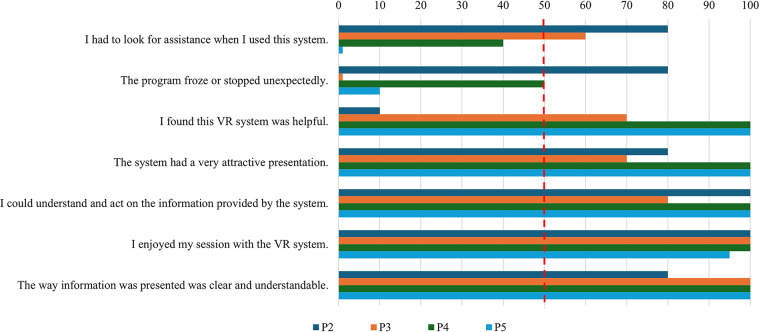
Bar charts illustrating the usability and satisfaction of the VR GMI program, as rated by each participant (*n* = 4). VR GMI, virtual reality graded motor imagery.

Four participants completed the entire VR GMI program over a range of three to five sessions in the postoperative acute care period for approximately 20 minutes each (*M =* 20.1 min, range = 6 — 45 min). The sociodemographic, clinical, and baseline characteristics of these participants, as well as their indications for LLA, are described in Table 1. See Table 2 for an overview of the VR GMI stages completed by each participant and reasons for termination. Overall impressions of the VR GMI program, including usability and satisfaction, can be reviewed in Figure 3.

**Table 1.** Participant Characteristics and Responses to VR GMI Intervention (*n* = 6)

ID	Age	Gender	POD at enrollment	Education	Amputation type	Indication	Prior VR use	PLP onset at enrollment	Physical comorbidities
1	18	Male	<1 week	Less than high school or GED equivalent	AKA	Cancer	Yes	No	History of multiple reconstructive surgeries, prosthetic infection, and prior chemotherapy
2	47	Male	1–4 weeks	Less than high school or GED equivalent	BKA	Nondiabetic ischemia	No	Yes	PVD; prior stroke
3	43	Male	<1 week	Postgraduate education	AKA	Cancer	Yes	Yes	None noted
4	69	Male	1–4 weeks	High school or GED equivalent	BKA	Diabetic	No	Yes	Type 2 diabetes mellitus; PVD
5	57	Female	4–8 weeks	High school or GED equivalent	Bilateral (BKA; midfoot)	Nondiabetic ischemia	Yes	Yes	Systemic scleroderma; PVD; interstitial lung disease
6	66	Male	<1 week	Less than high school or GED equivalent	BKA	Diabetic	Yes	Yes	Deep vein thrombosis; chronic kidney disease; chronic obstructive pulmonary disease; type 1 diabetes mellitus; PVD; hypertension

This table summarizes demographic, surgical, and clinical characteristics of all participants enrolled in the VR GMI program.

Information includes age, gender, POD of enrollment, education level, amputation type and indication, prior VR use, and presence of PLP at enrollment.

Physical comorbidities were extracted from medical charts to contextualize medical complexity.

AKA, above-knee amputation; BKA, below-knee amputation; GED, general educational development; PLP, phantom limb pain; POD, postoperative day; PVD, peripheral vascular disease; VR GMI, virtual reality graded motor imagery.

**Table 2.** Highest Stage Completed and Engagement in the VR GMI Program (*n* = 6)

Participant	Highest stage completed	# of sessions	Total VR time (min)	Reason for early termination or completion
P1	Left/right discrimination	1	10–20	Discharged home prior to further progression
P2	Limb simulation	5	90–100	Completed full VR GMI program
P3	Limb simulation	3	50–60	Completed full VR GMI program
P4	Limb simulation	3	60–70	Completed full VR GMI program
P5	Limb simulation	5	90–100	Completed full VR GMI program
P6	None	0	—	Difficulties with control manipulation during left/right discrimination; discharged before troubleshooting could occur

This table summarizes the highest stage completed by each participant in the VR GMI program.

Participants advanced sequentially through standardized stages, including left/right discrimination, explicit motor imagery, and limb simulation, with each preceding stage completed before progression.

Completion of the full program was defined as reaching the limb simulation stage.

VR GMI, virtual reality graded motor imagery.

#### Usability

The usability of the VR GMI program was measured through two items on the impressions questionnaire, including “I had to look for assistance when I used this system” and “The program froze or stopped unexpectedly,” as well as field notes indicating technical issues encountered during any session. Only three participants chose to respond to these items. Two participants indicated they had to look for assistance while using the system and that the program froze or stopped unexpectedly (as per ≥ 50% agreement). These ratings are corroborated by field notes, where participants often had to pass the headset back and forth with the RA to recenter the VR and optimize embodiment. Participant 1 (P1) stated: “*half the time the headset was on and it wasn’t working and I had to keep clicking; [the RA] had to take it off and figure it out.*” When the program froze or stopped, the RA assisted by manually resetting the headset. At times, the RA also took the controller to help participants navigate menus or select options when tremor, weakness, or poor tracking interfered with accurate pointing and clicking. These usability issues were typically resolved in real time without interrupting overall session flow.

#### Satisfaction

Satisfaction following short-term use of the VR GMI program in the hospital was assessed through five items on the impressions questionnaire and corroborated by field notes. Three out of four participants indicated that they found the VR GMI program helpful, and that they could understand and act on the information provided by the system. P1 endorsed low ratings for both items (0–10%). He cited having less than a high school education for his low comprehension of the system and indicated feeling fatigue in his phantom leg, which detracted from his perceived helpfulness of the VR. Other participants, such as P4, found the VR GMI program very helpful because it produced short-term relief in her PLP. Meanwhile, P2 endorsed the VR GMI program as slightly less helpful than the others (70%) because he was not permitted to continue using the VR GMI program following his hospital discharge. Otherwise, all four participants indicated they enjoyed their experience with VR GMI program, endorsed that the way the information was presented was clear and understandable, and thought the VR GMI program had a very attractive presentation (with endorsements ranging from 70% to 100% on these items).

#### Tolerability

P1, P2, P3, and P5 provided pre–post NRS scores indicating their tolerability of each VR GMI session (*n* = 2). The remaining participants did not report pre–post NRS scores (*n* = 2). P6 reported that he could not meaningfully assign a numerical value to his experience, and P4 chose to indicate qualitatively that he did not experience any increase or decrease in nausea, RLP, or PLP across sessions. Among participants who completed ratings, scores remained stable across sessions, with no notable changes in nausea. One participant (P2) reported a clinically significant increase in PLP of +7 points from pre- to postsession 5, which he endorsed as a clinically meaningful PLP increase described as fatigue. Another participant (P5) reported clinically significant decreases of −5 points in both RLP and PLP during session 2, and a smaller −3-point decrease in RLP during session 4. No other participants reported changes meeting the criterion for clinical significance. See Table 3 for a series of table depicting pre–post NRS scores for PLP, RLP, and nausea.

**Table 3.** Pre–Post NRS Scores for PLP, RLP, and Nausea in the VR GMI Program (*n* = 4)

NRS (0–10)	P1		P2		P3		P5	
	Pre	Post	Pre	Post	Pre	Post	Pre	Post
Session 1								
PLP	0	0	0	0	2	1	4	4
RLP	6	6	6	6	4	4	10	10
Nausea	0	0	0	0	0	0	0	0
Session 2								
PLP	NA		0	0	0	0	10	6*
RLP			7	7	3	3	6	2*
Nausea			1	1	0	0	0	0
Session 3								
PLP	NA		0	0	0	0	3	3
RLP			7	7	3	3	5	5
Nausea			0	0	0	0	0	0
Session 4								
PLP	NA		3	3	NA		7	5
RLP			8	8			7	4*
Nausea			0	0			0	0
Session 5								
PLP	NA		0	7*	NA		6	6
RLP			5	7			2	2
Nausea			0	0			0	0

NRS scores for PLP, RLP, and nausea before (pre) and after (post) each session of the VR GMI program.

Asterisks and color coding indicate clinically significant changes, defined as a ≥3-point difference between pre- and postsession scores.

Red shading denotes clinically significant increases in pain intensity, whereas blue shading denotes clinically significant decreases.

“NA” used where a session was not completed by a participant and thus did not provide NRS scores.

NRS, Numerical Rating Scale; PLP, phantom limb pain; RLP, residual limb pain; VR GMI, virtual reality graded motor imagery.

## Discussion

The current case series represents a proof-of-concept study for a novel VR GMI prototype administered in the postoperative acute care period as an early intervention/prevention for PLP immediately following LLA. Preliminary recruitment trends suggest low eligibility rates due to patient comorbidities precluding VR use such as severe diabetic neuropathy, retinopathy, impaired consciousness/cognition, critical illness, and major bilateral amputation. Moreover, many potential participants within the single research site at which recruitment was conducted were transferred or discharged within 3 days following their LLA, limiting time to engage in the VR GMI program under the current protocol. Fortunately, the few patients who were able to engage in the VR GMI program reported promising satisfaction and tolerability for short-term use of the intervention (i.e., 3–5 sessions) in the acute postoperative period. Only one patient experienced challenges with tolerability, reporting decreased satisfaction due to an unpleasant sensation of fatigue in the amputated limb. Conversely, observational and self-report measures demonstrate limited usability of the intervention in the acute postoperative period. Thus, further development of the VR GMI prototype should improve the accessibility of its context, controls, and content using a patient-centered approach to support implementation.

Early discharge, not commonly reported in prior studies, emerged as a significant barrier to participation in the current intervention delivered in the acute postoperative setting. For example, previous research investigating mirror therapy for PLP postoperatively in India did not identify discharge timing as a limitation.^5^ This discrepancy may reflect differences in hospital discharge practices across health care systems; Canada has relatively short average postoperative stays for individuals who do not require specialized or tertiary care following LLA, which may have contributed to the early discharge of several eligible individuals before they could engage in the VR GMI program.^33^ As a result, many individuals who remained hospitalized during the recruitment window may have had greater medical complexity, rendering them ineligible—consistent with previous research suggesting that the acute hospital setting is often associated with high rates of exclusion in medical extended reality interventions due to cognitive, motor, visual, or hearing impairment.^28^ The procedural difficulties associated with early hospital discharge may also reflect the nature of the VR GMI program itself. Unlike many hospital-based VR programs that use passive distraction and can be completed in a single session, the active VR GMI program requires multiple sessions to achieve a therapeutic effect.^22^ Although the current protocol allowed for VR GMI completion within 3 days, early discharge still occasionally limited full participation. Similar VR programs requiring longer administration have been successfully implemented in home settings, which may represent a more feasible delivery model for future applications of VR GMI.^18–20^ However, this would require further improvements to the system’s usability to support independent use outside the hospital. Together, these findings highlight that while acute inpatient delivery of VR GMI is feasible for select patients, early transition to home or rehabilitation settings may be necessary to ensure sufficient exposure and optimize therapeutic engagement.

Before the VR GMI program can be used independently by patients in their home, low usability ratings from the current study suggest barriers to access need to be mitigated. The prototype should be streamlined so it is accessible to people with cognitive, motor, and sensory impairment associated with diabetic LLA indications, given people with LLA are increasingly and disproportionately affected by diabetes in Canada.^33^ Potential VR GMI developments include introducing headphones to address hearing impairment, simplifying the hand controls so they are easier to hold and click or through the use of controllerless hand tracking, increasing text size, and limiting jargon. Moreover, a relatively large proportion of people with major bilateral amputations were excluded from participation; however, results from the current study suggest the VR GMI program can easily be adapted for use by this population. Given people with major bilateral amputations cannot benefit from mirror therapy and have previously been excluded from participating in similar VR interventions, subsequent studies will aim to improve the accessibility of the VR GMI program to this group.^15^ To address the educational needs of patients—particularly those with cognitive impairment—clinical experts recommend beginning GMI with thorough pain neuroscience psychoeducation (including visual diagrams to facilitate learning) to promote treatment buy-in, along with the creation of realistic and specific goals for daily engagement.^34^ To incorporate these recommendations, the next phase of VR GMI development will include embedded psychoeducation and emphasize 14–17 daily sessions of 20 min to see a treatment effect.^5^ These developments are intended to improve the accessibility and ultimately impact of the VR GMI program.

Beyond logistical barriers, psychological readiness appeared to play a key role in participation decisions. Several eligible patients declined due to feeling “overwhelmed,” which likely reflected the fatigue, pain, and emotional distress common in the immediate postoperative period.^14^ Qualitative work shows that early after amputation, patients often experience sedation, disorientation, and emotional shock that limit concentration and willingness to engage in new therapies.^24^ More broadly, hospitalized adults frequently cite fatigue, anxiety, and lack of perceived control as reasons for declining VR-based interventions.^28^ Together, these findings suggest that low uptake may reflect not only procedural barriers but also a mismatch between early recovery demands and the cognitive effort required for active VR. Expanding the eligibility window to within 2 months postamputation therefore aligned with existing evidence by maintaining the critical neuroplastic period for GMI while allowing additional time for medical stabilization and psychological readiness.^4^

Embedded psychoeducation within the VR GMI program may also be necessary to prime realistic expectations for the treatment course and improve long-term satisfaction. One participant in the current study reported decreased satisfaction with the intervention due to a short-term increase in nonpainful phantom fatigue following their sessions. Perhaps they would have been less discouraged had they been aware of previous research suggesting these effects can be expected following such exercises.^7,29^ A previous phase of development conducted in a community setting found personal VR treatment goals and expectations can have a large impact on satisfaction and buy-in; these findings extend to the postoperative acute care period.^24^ Unrealistic expectations for VR and treatment efficacy should be addressed as soon as possible to prevent dropout before the recommended dose can be administered. This could include a series of psychoeducation videos or audio clips embedded in the VR GMI program detailing common myths and barriers to use prior to administration. Building on this, using VR to assist with informed consent to prime expectations—as has outperformed standard approaches across surgical contexts, including orthopedics—could further enhance VR GMI engagement and may also help address the low uptake observed in the current study.^35^ Subsequent studies can elucidate the acceptability and usefulness of these embedded psychoeducation media to iteratively refine them, similar to the rest of the VR GMI program. This additional VR GMI content will be essential to improve user satisfaction, usability, and tolerability through realistic expectations.

In addition to managing expectations, tolerability findings suggest that accurate measurement of PLP represents another critical challenge for evaluating the VR GMI program in the immediate postoperative period. Transient fluctuations in PLP were observed before and after VR GMI sessions that appeared closely related to RLP, consistent with previous research suggesting these phenomena are highly correlated.^2^ However, two participants expressed confusion at being asked to quantify their experiences of PLP, RLP, and nausea to the extent that they chose not to report, calling into question the validity of the NRS to evaluate PLP in this population. Indeed, field notes indicated that sessions were occasionally ended early due to fatigue or discomfort in the phantom limb that was not captured by the NRS, suggesting that numeric pain ratings may not sufficiently capture short-term PLP in the postoperative context. In line with this insight, a recent review highlights the urgent need to validate standardized, multidimensional instruments that can differentiate between PLP and RLP in real time.^36^ For example, novel measurement techniques such as ecological momentary assessment utilize technology to provide a more granular longitudinal evaluation of chronic PLP intensity and interference.^37^ Accurate assessment in the postoperative period is even more complex, as PLP onset can occur anywhere from the first month to 1 year after amputation and is influenced by maturational bias.^3^ This trajectory underscores the importance of early intervention to treat or prevent PLP, while also highlighting the need for a control group to disentangle intervention effects from spontaneous recovery at longer-term follow-up beyond hospital discharge. A small research group from India administered mirror therapy during the immediate postoperative period and found a significant treatment effect on PLP intensity at 6-month follow-up, demonstrating the promise of this approach.^5^ Future investigations of the VR GMI program should similarly account for these methodological considerations when evaluating efficacy.

### Strengths and limitations

The primary strength of this case series rests in its application of the VR-CORE model, in an exploratory assessment of feasibility that will inform the subsequent execution of larger trials and eventual implementation of the VR GMI program.^25^ Qualitative field notes provide context to quantitative satisfaction, usability, and tolerability ratings in line with this patient-centered approach. Moreover, the present research was conducted at the largest tertiary hospital in Manitoba, where the majority of LLAs are conducted; thus, the low eligibility and consent rates identified should be representative of other sites in the province, given similar recruitment procedures.

However, recruitment feasibility must be interpreted with caution in light of mid-study protocol modifications. Initially, eligibility was restricted to individuals who had undergone an LLA within the past 5 days. This narrow window proved overly stringent, as many potential participants were discharged or transferred before they could be approached or complete the intervention. Expanding the inclusion window to within 2 months of surgery substantially improved recruitment and retention, while still leveraging evidence that PLP may be most amenable to intervention during this period.^4^ In addition, involving ward nurses and other health care team members in identifying and referring eligible patients during the final 2 months of recruitment resulted in the enrollment of the last three participants, highlighting the importance of staff engagement in feasibility protocols. Without these pragmatic changes, the study would likely not have been feasible. These adaptations were necessary to evaluate study procedures under realistic clinical conditions and to inform standardized recruitment strategies for the next, larger feasibility trial in accordance with the VR-CORE framework. However, these deviations from prespecified procedures may have introduced selection bias, as participants recruited later in their postoperative course could differ in recovery stage, clinical stability, or motivation compared with those initially eligible.

Another limitation of the current study is the representativeness of the final sample. As previously discussed, the current study was conducted in a unique Manitoban and Canadian context; thus, the generalizability of the results is limited, particularly given the case series design yielding a small sample size. Additionally, many Indigenous patients (a subpopulation in Manitoba disproportionally affected by diabetic LLA^38^) were transferred to Northern rural hospitals prior to trialling the VR GMI program, limiting even the provincial generalizability of this intervention. Nonetheless, the current case series fulfilled its purpose of demonstrating proof-of-concept for the VR GMI program.

Finally, several methodological limitations should be noted. Cognitive status was not formally assessed, as participants were screened informally for conversational ability rather than through standardized testing, which may limit interpretation of how cognitive variability influenced engagement or usability. Moreover, although PLP intensity was recorded as a measure of intervention tolerability, this outcome cannot be interpreted as a measure of efficacy without a control group. Postoperative PLP fluctuates considerably and is influenced by maturational bias (i.e., the natural improvement in pain and function that occurs during recovery independent of intervention effects). For similar reasons, individual analgesic regimens during VR GMI sessions were not systematically recorded, as they were outside the primary scope of this case series. Future controlled trials are needed to address these limitations.

### Implications

The current findings will contribute to the iterative development of the VR GMI program through a patient-centered approach to prepare it for an assessment of efficacy through larger sample sizes and more rigorous methodology. Lessons learned from the present study (including the need for optimized recruitment timing, providing upfront psychoeducation, and simplifying the user interface to be more accessible) will directly inform this next phase. Moreover, following early recruitment challenges, charge nurses were designated as the primary facilitators for identifying and referring eligible patients, which substantially improved recruitment efficiency. A large feasibility study will be conducted to assess the new VR GMI prototype with standardized methodology, which will include embedded psychoeducation media, refined recruitment strategies targeting patients later in their postoperative recovery, and validated measures to evaluate its acceptability, tolerability, and preliminary effectiveness as a PLP treatment. Focus groups with previous participants will be administered to refine and finalize the VR GMI program. Afterwards, a randomized controlled trial will be conducted to evaluate treatment effects. Once the feasibility and efficacy of the VR GMI program are established, implementation studies will be conducted to train inpatient rehabilitation physiotherapists in the administration and facilitation of the VR GMI program. Eventually, the VR GMI program could act as an early intervention or preventative measure for PLP, preventing negative downstream sequalae and improving quality of life for people with LLAs.^5,39^ The current case series represents one of the first steps toward delivering evidence-based PLP intervention to Canadians who need to access it.

## Conclusion

This case series demonstrated the preliminary feasibility of administering a VR GMI program in the acute postoperative period following LLA. Although challenges with recruitment and usability were encountered, these issues offered valuable insights into the practical realities of delivering a multisession VR intervention in a hospital setting. Importantly, the study identified procedural adaptations and prototype refinements—such as early engagement of nursing staff and simplified program controls—that will inform the next phase of development. Participants who engaged in the intervention generally reported high satisfaction and tolerability, reinforcing its potential acceptability during early recovery. Building on these findings, future research will explore recruitment for home-based use of the VR GMI program and the integration of embedded psychoeducation to improve usability and treatment engagement.

## Authors’ Contributions

Conceptualization: M.R., M.C., and R.E.-G. Data curation: M.C. and M.R. Formal analysis: M.R. and M.C. Funding acquisition: R.E.-G. and M.S. Investigation: M.C. and M.R. Methodology: M.R., M.C., and R.E.-G. Project administration: M.C., M.R., R.E.-G., and T.M. Resources: M.C., M.S., P.G., R.E.-G., E.H., and T.M. Software: M.S. Supervision: R.E.-G. and T.M. Validation: N/A. Visualization: M.C. Writing (original draft): M.C. and M.R. Writing (review and editing): M.C., M.R., R.E.-G., M.S., E.H., P.G., and T.M.

## Abbreviations

AKA: Above-knee amputation
BKA: Below-knee amputation
CONSORT: Consolidated Standards of Reporting Trials
GED: General educational development
GMI: Graded motor imagery
HSC: Health Sciences Center
LLA: Lower limb amputation
NRS: Numerical Rating Scale
PLP: Phantom limb pain
POD: Postoperative day
PVD: Peripheral vascular disease
RA: Research assistant
RLP: Residual limb pain
VR: Virtual reality
VR-CORE: Virtual Reality Clinical Outcomes Research Experts

## References

[1] Imam B, Miller WC, Finlayson HC, et al. Incidence of lower limb amputation in Canada. Can J Public Health, 2017; 108(4):e374–e380; doi: 10.17269/CJPH.108.6093 29120308 PMC6975214

[2] Mioton LM, Dumanian GA, Fracol ME, et al. Benchmarking residual limb pain and phantom limb pain in amputees through a patient-reported outcomes survey. Plast Reconstr Surg Glob Open, 2020; 8(7):e2977; doi: 10.1097/GOX.0000000000002977 32802669 PMC7413780

[3] Stankevicius A, Wallwork SB, Summers SJ, et al. Prevalence and incidence of phantom limb pain, phantom limb sensations and telescoping in amputees: A systematic rapid review. Eur J Pain, 2021; 25(1):23–38; doi: 10.1002/ejp.1657 32885523

[4] Agostinho M, Weissman Fogel I, Treister R. Time since onset might be of essence: A recommendation to assess the effects of combination of non-pharmacological neuromodulatory approaches at early stage since symptoms onset. Front Neurol, 2023; 14:1115370. 36793488 10.3389/fneur.2023.1115370 PMC9923174

[5] Purushothaman S, Kundra P, Senthilnathan M, et al. Assessment of efficiency of mirror therapy in preventing phantom limb pain in patients undergoing below-knee amputation surgery—A randomized clinical trial. J Anesth, 2023; 37(3):387–393; doi: 10.1007/s00540-023-03173-9 36809505

[6] Subedi B, Grossberg GT. Phantom limb pain: Mechanisms and treatment approaches. Pain Res Treat, 2011; 2011:864605; doi: 10.1155/2011/864605 22110933 PMC3198614

[7] Moseley GL, Butler DS, Beames TB, et al. The Graded Motor Imagery Handbook, 1st ed. Noigroup Publications: Adelaide, Australia; 2012.

[8] Wang J, Fan J, Gc R, et al. Comparative effects of interventions on phantom limb pain: A network meta-analysis. World Neurosurg, 2023; 170:e45–e56; doi: 10.1016/j.wneu.2022.10.060 36273725

[9] Moseley LG . Is successful rehabilitation of complex regional pain syndrome due to sustained attention to the affected limb? A randomised clinical trial. Pain, 2005; 114(1–2):54–61; doi: 10.1016/j.pain.2004.11.024 15733631

[10] Limakatso K, Cashin AG, Williams S, et al. The efficacy of graded motor imagery and its components on phantom limb pain and disability: A systematic review and meta-analysis. Can J Pain, 2023; 7(1):2188899; doi: 10.1080/24740527.2023.2188899 37214633 PMC10193907

[11] Liddy C, Cooper L, Bellingham G, et al. Patient-reported wait times and the impact of living with chronic pain on their quality of life: A waiting room survey in chronic pain clinics in Ontario, Manitoba, and Quebec. Can J Pain, 2024; 8(1):2345612; doi: 10.1080/24740527.2024.2345612 38894731 PMC11185187

[12] Johnson S, Hall J, Barnett S, et al. Using graded motor imagery for complex regional pain syndrome in clinical practice: Failure to improve pain. Eur J Pain, 2012; 16(4):550–561; doi: 10.1002/j.1532-2149.2011.00064.x 22337591

[13] Bakshi P, Chang W-P, Fisher TF, et al. Client buy-in: An essential consideration for graded motor imagery in hand therapy. J Hand Ther, 2021; 34(3):348–350; doi: 10.1016/j.jht.2020.03.014 32565107

[14] Alabdullah AAH, Alyazidi SS, Asiri IA, et al. A systematic literature review to explore lived experiences with phantom limb phenomenon following a lower extremity amputation: A qualitative synthesis. Front Rehabil Sci, 2025; 6:1667659; doi: 10.3389/fresc.2025.1667659 41050313 PMC12488712

[15] Cheung JC-W, Cheung DSK, Ni M, et al. X-reality for phantom limb management for amputees: A systematic review and meta-analysis. Eng Regen, 2023; 4(2):134–151; doi: 10.1016/j.engreg.2023.02.002

[16] Vassantachart AY, Yeo E, Chau B. Virtual and augmented reality-based treatments for phantom limb pain: A systematic review. Innov Clin Neurosci, 2022; 19(10–12):48–57. PMC9776775 36591552

[17] Lendaro E, Van der Sluis CK, Hermansson L, et al. Extended reality used in the treatment of phantom limb pain: A multicenter, double-blind, randomized controlled trial. Pain, 2025; 166(3):571–586; doi: 10.1097/j.pain.0000000000003384 39250328 PMC11808706

[18] Lendaro E, Middleton A, Brown S, et al. Out of the clinic, into the home: The in-home use of phantom motor execution aided by machine learning and augmented reality for the treatment of phantom limb pain. J Pain Res, 2020; 13:195–209; doi: 10.2147/JPR.S220160 32021409 PMC6983479

[19] Annapureddy D, Annaswamy TM, Raval G, et al. A novel mixed reality system to manage phantom pain in-home: Results of a pilot clinical trial. Front Pain Res, 2023; 4. 10.3389/fpain.2023.1183954 PMC10272374 37332478

[20] Munoz-Novoa M, van Veldhoven JE, Postema SG, et al. Regaining the intention to live after relief of intractable phantom limb pain: A case study. Scand J Pain, 2025; 25(1); doi: 10.1515/sjpain-2025-0006 40536824

[21] Schmitt YS, Hoffman HG, Blough DK, et al. Randomized, controlled trial of immersive virtual reality analgesia during physical therapy for pediatric burn injuries. Burns, 2011; 37(1):61–68; doi: 10.1016/j.burns.2010.07.007 20692769 PMC2980790

[22] Dascal J, Reid M, IsHak WW, et al. Virtual reality and medical inpatients: A systematic review of randomized, controlled trials. Innov Clin Neurosci, 2017; 14(1–2):14–21. 28386517 PMC5373791

[23] El-Gabalawy R, Crooks M, Smith MSD, et al. Treating lower phantom limb pain in the postoperative acute care setting using virtual reality: Protocol for a 4-phase development and feasibility trial. JMIR Res Protoc, 2025; 14(1):e68008; doi: 10.2196/68008 40409745 PMC12144477

[24] Crooks M, Smith M, Reynolds K, et al. Anticipated barriers and facilitators to engaging in a novel virtual reality program for lower phantom limb pain. J Med Ext Real, 2025; 2(1):61–75; doi: 10.1089/jmedxr.2024.0047 PMC13387243 42558182

[25] Birckhead B, Khalil C, Liu X, et al. Recommendations for methodology of virtual reality clinical trials in health care by an international working group: Iterative study. JMIR Ment Health, 2019; 6(1):e11973; doi: 10.2196/11973 30702436 PMC6374734

[26] Meek R, Egerton-Warburton D, Mee MJ, et al. Measurement and monitoring of nausea severity in emergency department patients: A comparison of scales and exploration of treatment efficacy outcome measures. Acad Emerg Med, 2015; 22(6):685–693; doi: 10.1111/acem.12685 25996342

[27] Rothaug J, Weiss T, Meissner W. How simple can it get? Measuring pain with NRS items or binary items. Clin J Pain, 2013; 29(3):224–232; doi: 10.1097/AJP.0b013e31824c5d7a 23369928

[28] Mosadeghi S, Reid MW, Martinez B, et al. Feasibility of an immersive virtual reality intervention for hospitalized patients: An observational cohort study. JMIR Ment Health, 2016; 3(2):e5801; doi: 10.2196/mental.5801 PMC4940605 27349654

[29] Mibu A, Nishigami T, Tanaka K, et al. Successful graded mirror therapy in a patient with chronic Deafferentation pain in whom traditional mirror therapy was ineffective: A case report. Pain Pract, 2016; 16(4):E62–E69; doi: 10.1111/papr.12431 26914841

[30] Sommer JL, Reynolds K, Hebbard P, et al. Preoperative virtual reality to expose patients with breast cancer to the operating room environment: Feasibility and pilot case series study. JMIR Form Res, 2024; 8:e46367; doi: 10.2196/46367 38231570 PMC10831694

[31] El-Gabalawy R, Sommer JL, Hebbard P, et al. An immersive virtual reality intervention for preoperative anxiety and distress among adults undergoing oncological surgery: Protocol for a 3-phase development and feasibility trial. JMIR Res Protoc, 2024; 13(1):e55692; doi: 10.2196/55692 38743939 PMC11134251

[32] Eldridge SM, Chan CL, Campbell MJ, et al. CONSORT 2010 statement: Extension to randomised pilot and feasibility trials. BMJ, 2016:i5239; doi: 10.1136/bmj.i5239 27777223 PMC5076380

[33] Canadian Institute for Health Information. Inequalities in diabetes-associated lower limb amputations. CIHI; 2024. Available from: https://www.cihi.ca/en/equity-in-diabetes-care-a-focus-on-lower-limb-amputation/inequalities-in-diabetes-associated-lower-limb-amputations [Last accessed: May 5, 2025].

[34] Rich TL, Marth LA, Brielmaier SM, et al. Strategies for graded motor imagery for clients with phantom limb pain and cognitive impairment. Prosthet Orthot Int, 2022; 46(5):496–499; doi: 10.1097/PXR.0000000000000125 35333828

[35] Wehrkamp K, Miksch RC, Polzer H, et al. The impact of virtual-, augmented- and mixed reality during preoperative informed consent: A systematic review of the literature. J Med Syst, 2025; 49(1):89; doi: 10.1007/s10916-025-02217-9 40555846 PMC12187789

[36] Jenson AN, Branch B, Richard JM, et al. Phantom limb pain assessment tools: A literature review exploring strengths and limitations. Arch Rehabil Res Clin Transl, 2025; 7(2):100453; doi: 10.1016/j.arrct.2025.100453 40678285 PMC12265911

[37] May M, Junghaenel DU, Ono M, et al. Ecological momentary assessment methodology in chronic pain research: A systematic review. J Pain, 2018; 19(7):699–716; doi: 10.1016/j.jpain.2018.01.006 29371113 PMC6026050

[38] Blanchette V, Patry J, Brousseau-Foley M, et al. Diabetic foot complications among Indigenous peoples in Canada: A scoping review through the PROGRESS-PLUS equity lens. Front Endocrinol, 2023; 14; doi: 10.3389/fendo.2023.1177020 PMC10461566 37645408

[39] Sinha R, Van Den Heuvel WJA, Arokiasamy P, et al. Influence of adjustments to amputation and artificial limb on quality of life in patients following lower limb amputation. Int J Rehabil Res, 2014; 37(1):74–79; doi: 10.1097/MRR.0000000000000038 24157864

